# Molecular Diversity and Combining Ability in Newly Developed Maize Inbred Lines under Low-Nitrogen Conditions

**DOI:** 10.3390/life14050641

**Published:** 2024-05-17

**Authors:** Mohamed M. Kamara, Elsayed Mansour, Ahmed E. A. Khalaf, Mohamed A. M. Eid, Abdallah A. Hassanin, Ahmed M. Abdelghany, Ahmed M. S. Kheir, Ahmed A. Galal, Said I. Behiry, Cristina Silvar, Salah El-Hendawy

**Affiliations:** 1 Department of Agronomy, Faculty of Agriculture, Kafrelsheikh University, Kafr El-Sheikh 33516, Egypt; mohamed.kamara@agr.kfs.edu.eg (M.M.K.); aa.galal79@gmail.com (A.A.G.); 2 Department of Crop Science, Faculty of Agriculture, Zagazig University, Zagazig 44519, Egypt; sayed_mansour_84@yahoo.es; 3 Agronomy Department, Faculty of Agriculture, Fayoum University, Fayoum 63514, Egypt; asa14@fayoum.edu.eg (A.E.A.K.); mam11@fayoum.edu.eg (M.A.M.E.); 4 Department of Genetics, Faculty of Agriculture, Zagazig University, Zagazig 44519, Egypt; dr.abdallah4@gmail.com; 5 Crop Science Department, Faculty of Agriculture, Damanhour University, Damanhour 22516, Egypt; ahmed.abdelghany@agr.dmu.edu.eg; 6 Soils, Water and Environment Research Institute, Agricultural Research Center, Giza 12619, Egypt; drahmedkheir2015@gmail.com; 7 Agricultural Botany Department, Faculty of Agriculture (Saba Basha), Alexandria University, Alexandria 21531, Egypt; said.behiry@alexu.edu.eg; 8 Grupo de Investigación en Bioloxía Evolutiva, CICA—Centro Interdisciplinar de Química e Bioloxía, Universidade da Coruña, 15071 A Coruña, Spain; c.silvar@udc.es; 9 Department of Plant Production, College of Food and Agriculture Sciences, King Saud University, P.O. Box 2460, Riyadh 11451, Saudi Arabia

**Keywords:** nitrogen deficiency, genetic diversity, hybrid breeding, microsatellite markers, sustainable agriculture

## Abstract

Nitrogen is an essential element for maize growth, but excessive application can lead to various environmental and ecological issues, including water pollution, air pollution, greenhouse gas emissions, and biodiversity loss. Hence, developing maize hybrids resilient to low-N conditions is vital for sustainable agriculture, particularly in nitrogen-deficient soils. Combining ability and genetic relationships among parental lines is crucial for breeding superior hybrids under diverse nitrogen levels. This study aimed to assess the genetic diversity of maize inbred lines using simple sequence repeat (SSR) markers and evaluate their combining ability to identify superior hybrids under low-N and recommended conditions. Local and exotic inbred lines were genotyped using SSR markers, revealing substantial genetic variation with high gene diversity (He = 0.60), moderate polymorphism information content (PIC = 0.54), and an average of 3.64 alleles per locus. Twenty-one F1 hybrids were generated through a diallel mating design using these diverse lines. These hybrids and a high yielding commercial check (SC-131) were field-tested under low-N and recommended N conditions. Significant variations (*p* < 0.01) were observed among nitrogen levels, hybrids, and their interaction for all recorded traits. Additive genetic variances predominated over non-additive genetic variances for grain yield and most traits. Inbred IL3 emerged as an effective combiner for developing early maturing genotypes with lower ear placement. Additionally, inbreds IL1, IL2, and IL3 showed promise as superior combiners for enhancing grain yield and related traits under both low-N and recommended conditions. Notably, hybrids IL1×IL4, IL2×IL5, IL2×IL6, and IL5×IL7 exhibited specific combining abilities for increasing grain yield and associated traits under low-N stress conditions. Furthermore, strong positive associations were identified between grain yield and specific traits like plant height, ear length, number of rows per ear, and number of kernels per row. Due to their straightforward measurability, these relationships underscore the potential of using these traits as proxies for indirect selection in early breeding generations, particularly under low-N stress. This research contributes to breeding nitrogen-efficient maize hybrids and advances our understanding of the genetic foundations for tolerance to nitrogen limitations.

## 1. Introduction

Maize (*Zea mays* L.) is a globally significant staple crop vital for human and animal food [1,2]. Its wide adaptability and nutritional value have led to its cultivation in diverse agroecological zones worldwide [3]. Moreover, maize finds applications in various industries, including biofuel production, pharmaceuticals, and manufacturing [4]. The global acreage of maize is around 203 × 10^6^ hectares, which produces approximately 1163 × 10^6^ tons of grains [1]. In many countries, maize is an important source of national income [5,6]. With its growing population and increasing demand for maize, Egypt faces a notable production–consumption gap of around nine million tons annually [1]. Despite efforts to enhance production, the country heavily relies on imports to meet its maize requirements. This dependence is not sustainable in the long run and poses economic challenges. Maize production and productivity in Egypt are severely constrained by several factors, including unpredictable climatic conditions, low soil nitrogen (low-N), and the emergence of new insect pests or diseases. Developing high-yielding and stress-resilient maize hybrids should be a top priority to overcome these challenges and enhance maize productivity. These efforts will contribute to overcoming the existing constraints and establishing sustainable maize production practices in the country.

Nitrogen (N) serves as the most critical nutrient factor in sustaining high on-farm maize yields. Among all crops globally, maize is a significant nitrogen consumer, utilizing almost a fifth of the total nitrogen produced worldwide [7,8]. The excessive application of nitrogen fertilizers results in economic losses and environmental degradation, including soil acidification and the pollution of water bodies and the atmosphere [9]. Adequate nitrogen supply is essential for the recommended growth, development, and productivity. It is essential for various physiological processes in plants, such as chlorophyll production, photosynthesis, protein synthesis, and enzyme activities [10,11]. The detrimental effects of low-N availability on maize grain yield and its components have been well-documented in various studies [12,13,14,15]. Nitrogen deficiency can result in stunted plant growth, decreased leaf area, and reduced photosynthetic efficiency, contributing to lower grain yields [16]. Furthermore, low nitrogen levels can adversely affect grain yield components, such as kernel number and weight, thereby reducing the overall yield [17,18].

Understanding the combining ability of maize inbred lines is paramount for developing high-yielding and low-N tolerance hybrids. Identifying suitable parental inbred lines with superior genetic traits enables maize breeders to select combinations that strategically maximize heterosis or hybrid vigor [19,20,21,22]. Exploring general and specific combining abilities (GCA and SCA) and the inheritance patterns of agronomic traits is essential to develop hybrids with diverse genetic backgrounds and enhance resilience to environmental stresses. The diallel mating design is an effective method to investigate the effects of specific and general combining abilities. Furthermore, this method effectively understands genetic information that controls the inheritance of targeted traits through early generations [23,24,25]. The analysis of diallel design can be applied using Griffing’s method, which explores GCA and SCA [23,25,26]. Furthermore, this analysis can be employed to study the additive and non-additive effects of studied traits [27,28,29]. SCA is related to dominance effects; however, GCA is ascribed to additive impacts [30]. Numerous studies investigated how yield traits in maize are genetically controlled under various nitrogen conditions. Non-additive and additive gene actions are crucial for yield contributing trait inheritance in recommended conditions [31,32,33]; however, non-additive effects predominantly influence these traits under low nitrogen soil levels [14,34,35]. Other studies presented contrasting evidence, indicating additive gene actions as the primary influence on grain yield inheritance under nitrogen-deficient conditions [36,37,38]. These discrepancies could arise from the different levels of nitrogen stress applied during the evaluation of the hybrids.

Inbred lines serve as fundamental genetic resources for maize improvement efforts. Understanding the extent and distribution of genetic diversity among maize inbred lines is crucial for effective breeding strategies to develop promising hybrid and introgression favorable alleles to enhance crop productivity and resilience [14]. Molecular genetic diversity is pivotal for rapidly identifying robust hybrids without testing every possible parental combination in breeding initiatives [29]. This diversity encompasses variations in morphological, agronomic traits and molecular markers reflecting genomic differences. Thus, elucidating the genetic diversity of maize inbred lines is indispensable for developing new high-yielding hybrids to sustain maize production and food security [31,32,39,40]. Recent advances in DNA markers play a crucial role in revealing the genetic distances between maize inbreds. Simple Sequence Repeats (SSRs) are highly valued for their informativeness, multi-allelic nature, co-dominance, and reproducibility [3]. The evaluated maize inbred lines under study were hypothesized to demonstrate significant genetic variability, laying a foundation for developing superior maize hybrids under low nitrogen conditions in arid environments. Thus, the present study aimed to (i) explore the genetic distances between exotic and local maize inbreds employing SSR markers; (ii) assess the agronomic performance of F1 hybrids under recommended (288 kg N ha^−1^) and low-N (192 kg N ha^−1^) stress conditions; (iii) analyze GCA and SCA for the traits under examination; and (iv) investigate the interrelationship among traits under both recommended and low-N stress conditions.

## 2. Materials and Methods

### 2.1. Plant Materials and Experimental Design

**Figure 1:**
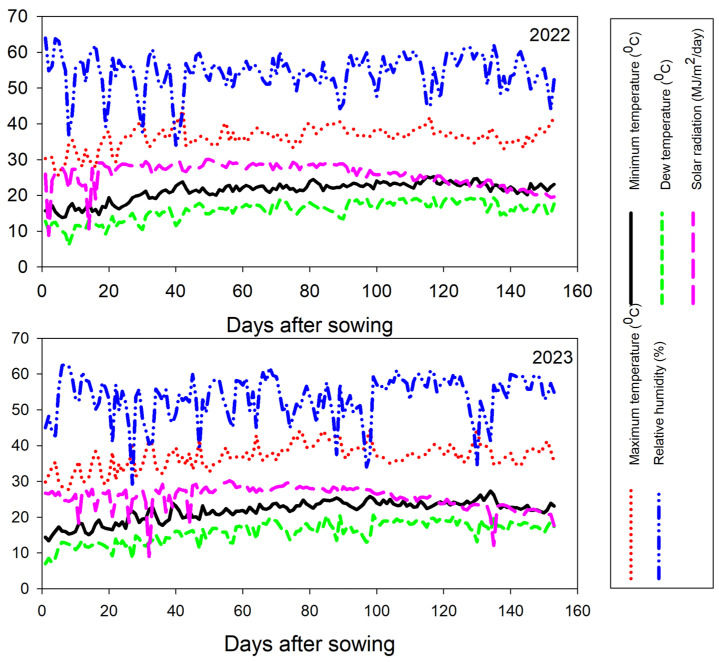
The minimum and maximum temperatures along with solar radiation levels, were recorded across the growing seasons.

Seven diverse maize inbreds (*Zea mays* L.) were utilized in the current study. The pedigree and source of these inbreds are given in Table S1. The used inbred lines were hybridized through adopting a half-diallel mating scheme, and 21 F1 single crosses were developed in the summer season of 2022. The developed F_1_ hybrids and commercial high-yielding hybrid SC-131 were evaluated under two nitrogen levels (288 and 192 kg N ha^−1^) during the summer season of 2023 at El-Mahmoudia, El-Behira, Egypt (31°3′ N 30°48′ E). The recommended nitrogen dose under Egyptian conditions for maize cultivation under the study area is 288 kg N ha^−1^. This amount was diminished by 35% to provide low nitrogen conditions by adding 192 kg N ha^−1^. The trials took place in an area known for its dry and hot climate since Egypt experiences no rainfall during the summer (Figure 1). The site soil is clay, according to the World Reference Base for Soil Resources [41], with the following profile: 13.2% sand, 33.2% silt, and 53.6% clay. The soil characteristics of the experimental site are detailed in Table S2. The employed experimental design was a split-plot design with three replications. Nitrogen levels were randomized across the main plots, while the hybrids were randomized within the subplots. Maize seeds were sown in two rows plots, each 6 m long, maintaining 0.70 m between rows and 0.25 m between plants within rows. The seedlings were thinned to one per hill 21 days post-planting to achieve the recommended plant density. Furrow irrigation was applied following the practice of the region of the study. The irrigation occurred at 11–13 day intervals, providing around 8000 m^3^/ha over the growing season. This application aligns with the recommendation of the Department of Water Requirement and Field Irrigation, part of the Center of Agricultural Research under the Egyptian Ministry of Agriculture and Land Reclamation. Nutrient management included the application of phosphorus (as calcium superphosphate with 15.5% P_2_O_5_) at a rate of 76 kg P_2_O_5_ per hectare during seedbed preparation and potassium sulfate (48% K_2_O) at 116 kg K_2_O per hectare post-thinning. Nitrogen, in the form of urea, was administered in two equal doses aligned with the first and second irrigations. Standard agronomic practices including insect pest and weed control were applied as recommended for growing maize in the region.

### 2.2. Data Collection

The agronomic traits were determined at physiological maturity. Plant height (PH, measured in centimeters) was determined by measuring the distance from the ground to the apex of the first tassel branch. Similarly, ear height (EH, in centimeters) was determined as the distance from the ground level to the base of the highest ear. Days to Silking (DS) was recorded when half of the plants within each plot began to produce silks, indicating the onset of the reproductive phase. During the harvest, a randomized selection of ten ears from each plot was used to evaluate agronomic traits, including the number of kernels per row (NK/R), ear length (EL), the number of rows per ear (NR/E), and weight of 1000 kernels (TKW). Plots were hand-harvested, and the weight of the shelled grain (adjusted to 15.5% grain moisture content) was used to calculate grain yield (t/ha)

### 2.3. Statistical Analysis

Analysis of variance (ANOVA) was computed for all data using SAS software (version 9.1). The combining abilities were analyzed following Griffing’s method 4, model 1 [42]. The least significant difference (*p* < 0.05) test was implemented to display the significance of variations among means. The analyses of principal components and the heatmap were generated based on averages of the tested traits to explore their associations. In addition, the hierarchical cluster analysis was applied to group the evaluated maize hybrids based on the studied yield traits using R statistical programming software version 4.2.1.

### 2.4. Molecular Analysis

DNA was extracted from the fresh leaves (200 mg) of maize inbred lines using the cetyltrimethylammonium bromide (CTAB) method [43]. The quality and concentration of the extracted DNA were assessed using a NanoDrop spectrophotometer. For the genotyping analysis (ND-1000, NanoDrop Technologies, Wilmington, NC, USA), twenty-one Simple Sequence Repeat (SSR) primer pairs were selected, with their sequences listed in Table S3, as sourced from the Maize Genome Database (Maize-GDB). The polymerase chain reaction (TaKaRa PCR Thermal Cycler TP650, Kusatsu, Japan) was performed in a reaction mixture of ten μL, comprising one μL of genomic DNA (20 ng/μL), 0.2 mM of dNTPs, two mM of MgCl_2_, 0.5 μM of each forward and reverse primer and 1 unit of Taq DNA polymerase. The PCR protocol initiated with a pre-denaturation stage at 94 °C for 2 min, then denaturation at 94 °C for 30 s, annealing at 55 °C for 30 s, and finally, extension at 72 °C for 30 s. The elongation step was conducted at 72 °C for 3 min. The PCR products were subsequently separated on a 1.5% agarose gel and visualized. The gels underwent staining with ethidium bromide, were destained using tap water, and then photographed using a gel documentation system (UVITEC, Cambridge, UK). The presence or absence of specific bands for each SSR marker was recorded, generating a binary data matrix coded as (0) for absence and (1) for presence. The analysis was performed using the PAST software (version 2.17) to calculate the genetic distances among the parental inbred lines, adhering to the methodology described by Jaccard [44].

## 3. Results

### 3.1. Genetic Diversity among Inbred Lines

**Figure 2:**
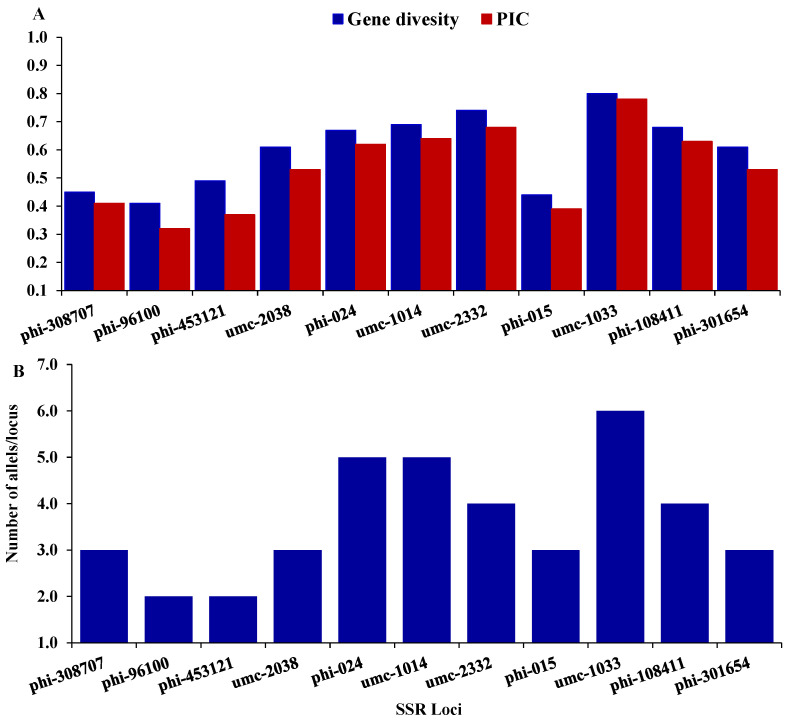
(**A**) depicts gene diversity and Polymorphic Information Content (PIC) for each marker, and (**B**) illustrates the number of alleles per locus.

Twenty-one SSR markers were employed to distinguish the genetic variability among seven maize inbreds. Out of them, eleven SSR markers were detected to be polymorphic. These polymorphic SSR markers were employed to explore the genetic variability among the tested inbreds in the present study. The number of alleles per locus altered from 2 (phi-453121, and phi-9610) to 6 (umc-1033), with an average of 3.64 alleles/locus (Figure 2A). The gene variability differed from 0.41 to 0.8, averaging 0.60 (Figure 2B). The average polymorphic information content (PIC) was 0.54, varying from a low of 0.32 (phi-96100) to a high of 0.78 (umc-1033).

**Figure 3:**
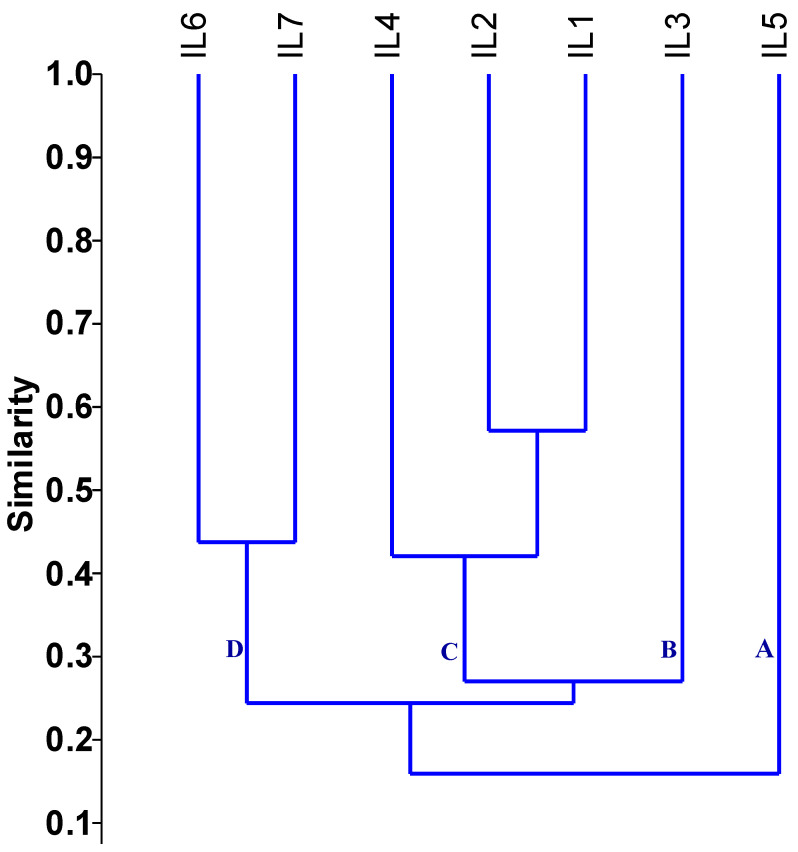
Dendrogram of the assessed inbreds based on applied SSR markers. The evaluated inbred lines were grouped into four primary clusters, each with varying levels of genetic diversity.

The genetic distance assessed using SSR markers fluctuated from 0.43 to 0.88, with an average of 0.73, as shown in Table 1. The minimum genetic distance was observed between inbred lines IL1 and IL2 (0.43). In contrast, the maximum genetic distance of 0.88 occurred between the inbred lines (IL1 and IL5), (IL2 and IL5), and (IL4 and IL5). The dendrogram constructed based on the genetic distance matrix grouped the evaluated inbred lines into four primary clusters, each with varying levels of genetic diversity (Figure 3). Group A consisted only of IL5 and group B contained IL3. Group C contained three lines divided into two subgroups: the first subgroup included IL1, IL2, while the second subgroup comprised IL4. Group D included IL6 and IL7.

### 3.2. Analysis of Variance

The analysis of variance displayed that the nitrogen level (N), developed hybrids (H), and their interaction (H×N) significantly influenced all studied agronomic traits (Table 2). Both general (GCA) and specific (SCA) combining abilities showed significant mean squares across all studied traits. However, the interaction effects of GCA×N and SCA×N were significant for most traits, with the exceptions of GCA×N for days to silking and number of rows per ear, and SCA×N for plant height, which were not significant. The ratio of GCA to SCA exceeded unity for all traits, suggesting a dominance of additive genetic effects (Table 2 and Table S4). Additionally, the interaction effect of GCA×N was greater than that of SCA×N for most traits, except for days to silking, ear height, and ear length, as noted in Table 2.

### 3.3. Performance of the Evaluated Hybrids

**Figure 4:**
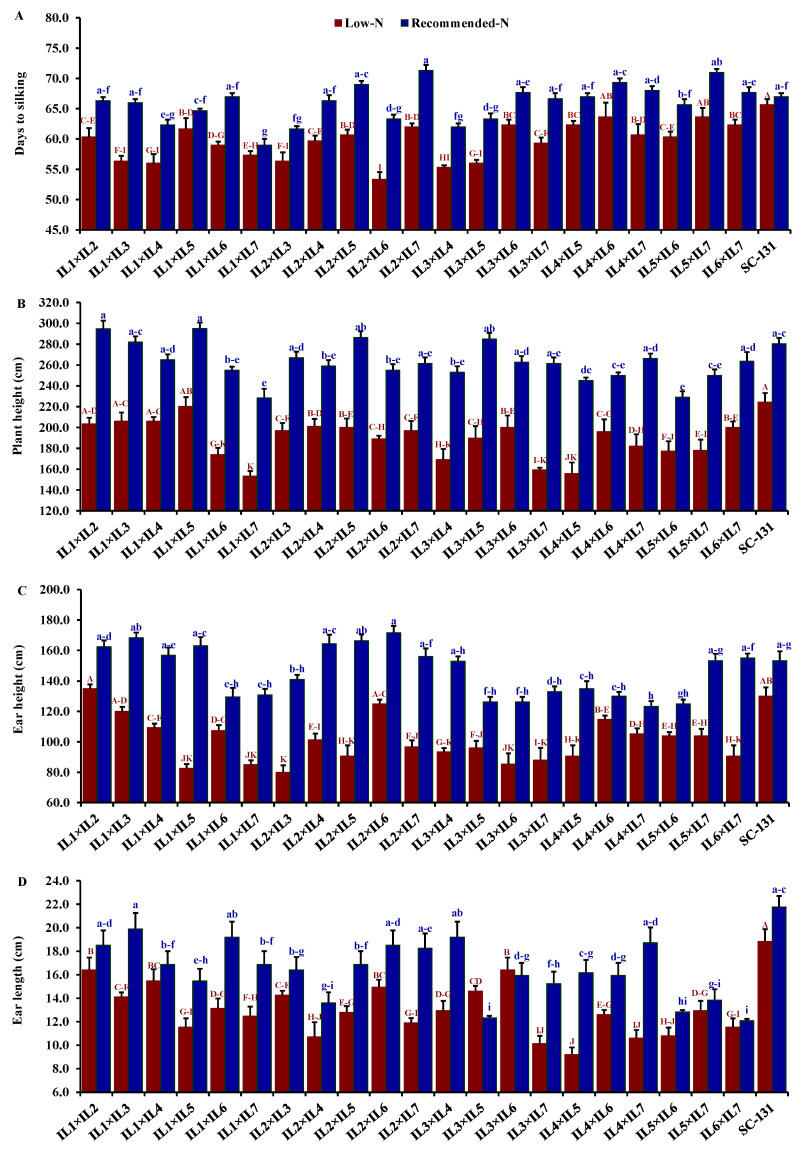
Comparative performance of developed 21 F1 hybrids and check hybrid (SC-131): 1 days to silking (**A**), plant height (**B**), ear height (**C**), and ear length (**D**). The bars on the tops of the columns represent the SE, and different letters on the columns show the significant difference using the Least Significant Difference (LSD) (*p* < 0.05). The uppercase letters belong to low N while the lowercase letters belong to the recommended conditions.

**Figure 5:**
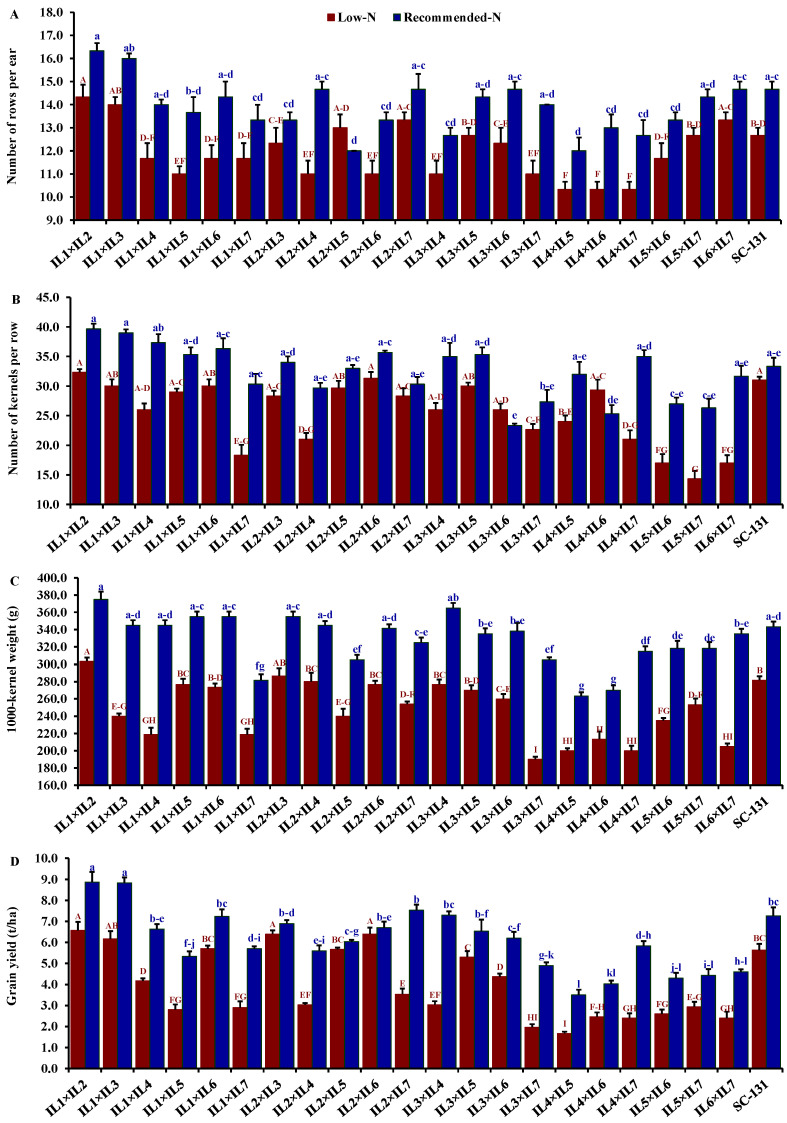
Comparative performance of developed 21 F1 hybrids and check hybrid (SC-131): number of rows per ear (**A**), number of kernels per row (**B**), 1000-kernel weight (**C**), and grain yield (**D**). The bars on the tops of the columns represent the SE, and different letters on the columns show the significant difference using the Least Significant Difference (LSD) (*p* < 0.05). The uppercase letters belong to low N while the lowercase letters belong to the recommended conditions.

Nitrogen levels significantly influenced all evaluated traits, with low-N stress markedly reducing key agronomic measurements. Under low-N conditions compared to recommended levels, days to silking decreased by 9.5%, plant height by 28.0%, ear height by 30.7%, ear length by 19.5%, number of rows/ear by 13.9%, number of kernels/row by 21.1%, thousand kernel weight by 24.6%, and grain yield by 32.1%. The hybrids exhibited considerable genetic diversity under both low-N and recommended conditions. Days to silking varied from 55.3 to 65.7 days (average 61.5 days) under low-N stress and from 59.0 to 71.3 days (average 66.0 days) under recommended conditions. The hybrids IL1×IL7 and IL2×IL6 flowered earliest, whereas IL5×IL7 and IL2×IL7 were the latest under their respective conditions (Figure 4A). Plant height averaged 188.3 cm under low-N, ranging from 153.3 to 220.3 cm, and 262.6 cm under recommended conditions, ranging from 228.3 to 295 cm. IL1×IL5 was consistently the tallest, and IL1×IL7 the shortest under both conditions (Figure 4B). The ear height showed similar variability, with means of 100.2 cm under low-N and 146.3 cm under recommended conditions, ranging from 80.0 to 135.0 cm and 123.3 to 171.7 cm, respectively (Figure 4C). IL1×IL2 and IL2×IL6 had the highest ear placements under low-N and recommended conditions, respectively, while IL2×IL3 and IL4×IL7 had the lowest. Ear length, under stress, ranged from 9.20 to 16.4 cm and from 12.1 to 19.9 cm under normal conditions. Hybrids IL3×IL6 and IL1×IL3 exhibited the longest ears under each condition, respectively (Figure 4D). The number of rows per ear varied from 10.3 to 14.3 (average 11.9) under low-N and 12.0 to 16.3 (average 13.9) under recommended conditions. Hybrids IL1×IL2 and IL1×IL3 produced the maximum number of rows, while IL4×IL5 had the fewest (Figure 5A). The number of kernels per row averaged 25.3 under low-N and 32.3 under recommended conditions, with IL1×IL2 showing the highest counts and IL5×IL7 and IL3×IL6 the lowest under their respective conditions (Figure 5B). The thousand-kernel weight varied from 190.0 to 303.3 g (average 246.3 g) under low-N and from 263.3 to 375.0 g (average 328.2 g) under recommended conditions, with IL3×IL7 showing the lightest and IL1×IL2 the heaviest weights under both conditions (Figure 5C). Finally, grain yield varied from 1.7 to 6.6 t ha^−1^ (average 4.0 t/ha) under low-N and from 3.5 to 8.9 t/ha (average 5.9 t/ha) under recommended conditions. The highest yields under low-N were assigned for hybrids IL1×IL2, IL2×IL6, and IL3×IL5, while under recommended conditions, the highest yields were from IL1×IL2, IL1×IL3, and IL2×IL7 (Figure 5D).

### 3.4. Classification of Hybrids

**Figure 6:**
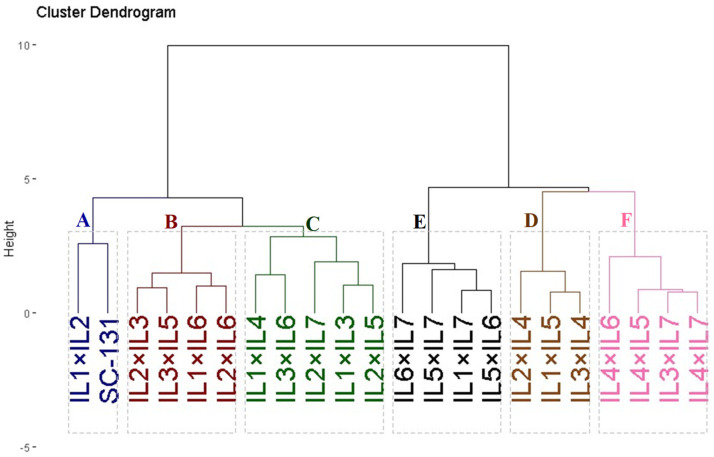
Dendrogram of phenotypic distances among twenty-one hybrids and commercial check SC-131 based on the yield traits. The evaluated twenty-one F1 maize hybrids and the commercial check SC-131 were clustered based on yield characteristics into six groups. Group A exhibited the highest yield and related traits while group F showed the lowest agronomic performance.

Twenty-one developed F1 maize hybrids and the commercial check SC-131 were clustered based on yield characteristics such as the number of rows per ear, ear length, 1000-kernel weight, kernels per row, and grain yield. These hybrids were divided into six groups using hierarchical clustering, as depicted in Figure 6. Group A, comprising two hybrids (IL1×IL2 and SC-131), exhibited the highest yield and related traits. Group B included four hybrids (IL2×IL3, IL3×IL5, IL1×IL6, and IL2×IL6) characterized by high yield traits. Groups C and D contained five and three hybrids and showed moderate yield traits. Conversely, Groups E and F encompassed hybrids with the lowest agronomic performance.

### 3.5. General Combining Ability (GCA)

All evaluated agronomic traits exhibited strong positive general combining ability (GCA) effects except for days to silking, plant height and ear heights. According to Table 3, the inbred lines IL1 and IL3 showed the most significant negative GCA effects for days to silking, whereas IL7 had notably negative GCA effects on plant height under low-N and recommended conditions. IL3 and IL7 were identified as the best combiners for ear height, displaying negative and desirable GCA effects under both conditions. Regarding ear length, IL1, IL2, and IL3 demonstrated the highest positive GCA effects under low-N conditions, with IL1 and IL2 also showing significant positive effects under recommended conditions. Additionally, IL1 and IL2 under low-N and IL1 under recommended conditions had notably positive GCA effects for the number of rows/ear. The strongest GCA effects for the number of kernels per row were attributed to IL1, IL2, and IL3 under low-N and to IL1 and IL2 under recommended conditions. Moreover, inbred lines IL1, IL2, and IL3 consistently exhibited the most substantial positive GCA effects for thousand-kernel weight and grain yield across low-N and recommended conditions.

### 3.6. Specific Combining Ability (SCA)

The assessed hybrids exhibited diverse specific combining ability (SCA) effects across all studied traits. For days to silking, the crosses IL1×IL4 under low-N, IL5×IL6, and IL2×IL3 under recommended conditions, along with IL1×IL7, IL2×IL6, IL3×IL4, and IL3×IL5 under both conditions, demonstrated significant negative effects (Table 4). These hybrids could be utilized in maize breeding programs to promote earliness. Significant negative SCA effects for plant height were recorded in hybrids such as IL1×IL6, IL1×IL7, IL3×IL4, IL3×IL7, and IL4×IL5 under low-N conditions, and IL1×IL7, IL2×IL3, IL4×IL5, and IL5×IL6 under optimal conditions. For ear height, hybrids like IL1×IL5, IL1×IL7, IL2×IL3, and IL3×IL6 under low-N, and IL1×IL6, IL1×IL7, IL2×IL3, IL3×IL5, IL4×IL7, and IL5×IL6 displayed significantly negative SCA effects. Otherwise, the most significant positive SCA effects for ear length were exhibited by IL1×IL4, IL2×IL6, IL3×IL5, IL5×IL7, and IL3×IL5 under low-N, and IL1×IL3, IL1×IL6, IL2×IL5, IL2×IL6, IL2×IL7, IL3×IL4, IL4×IL5, and IL4×IL7 under recommended conditions. The highest positive effects for the number of rows per ear were noted in hybrids IL1×IL2, IL1×IL3, and IL6×IL7 under low-N, and IL1×IL2, IL1×IL3, IL2×IL4, IL3×IL5, and IL5×IL7 under recommended conditions. Additionally, the crosses IL2×IL7, IL3×IL5, and IL4×IL6 had the highest positive effects for the number of kernels per row under low-N, whereas IL2×IL6, IL3×IL5, IL4×IL7, and IL6×IL7 demonstrated the strongest effects under recommended conditions. Seven and nine hybrids showed significant positive SCA effects for the thousand-kernel weight under low-N and recommended conditions, respectively, with the most desirable effects attributed to IL1×IL2, IL1×IL5, IL1×IL6, IL2×IL4, IL3×IL4, and IL5×IL7 across both conditions. For grain yield, the hybrids IL1×IL4, IL2×IL5, IL2×IL6, and IL5×IL7 under low-N, IL1×IL2, IL2×IL7, and IL3×IL4 under recommended conditions, along with IL1×IL3, IL1×IL6, IL3×IL5, and IL4×IL7 under both conditions, displayed significant positive SCA effects. Notably, no hybrids demonstrated favorable SCA effects for all studied traits simultaneously. However, specific hybrids like IL1×IL3 and IL1×IL6 showed beneficial effects for grain yield and other yield-related traits, proving to be effective specific combiners under low-N and optimal conditions.

### 3.7. Interrelationship among Evaluated Hybrids and Measured Traits

**Figure 7:**
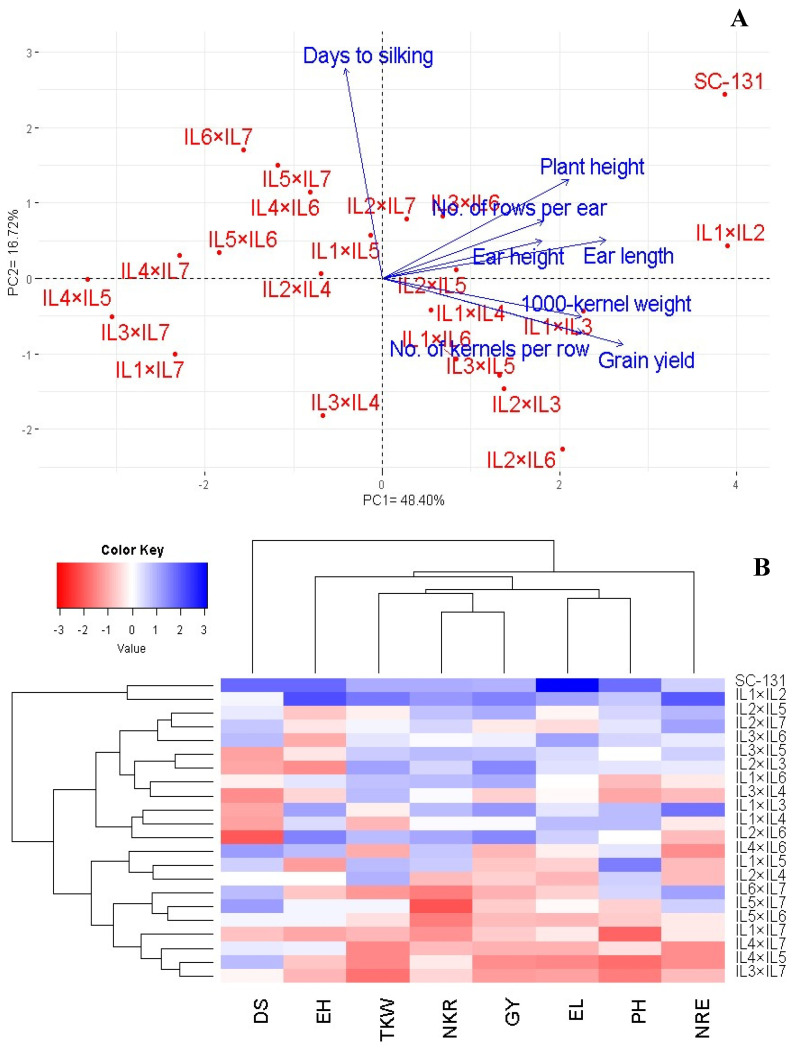
PC-biplot (**A**) and heatmap and hierarchical clustering (**B**) were used to study the association between agronomic traits and develop maize hybrids. PH: plant height; DS: days to silking; EL: ear length; NRE: number of rows/ear; NKR: number of kernels/row; GY: grain yield; and TKW: 1000-kernel weight.

The relationship between the assessed hybrids and their agronomic traits was explored through principal component analysis (PCA). The first two principal components accounted for a significant portion of the variance (48.40% by PC1 and 16.72% by PC2), and were visualized in a PC-biplot (Figure 7A). PC1 showed a higher variation and effectively differentiated the hybrids into those with positive and negative values on this axis. The hybrids on the positive side of PC1 were associated with superior agronomic traits, particularly notable in IL1×IL2 and SC-131. Conversely, hybrids on the negative side, such as IL4×IL5, IL3×IL7, and IL4×IL7, displayed lower agronomic performance. The proximity of vectors in the biplot indicated a strong positive correlation among the traits. Specifically, grain yield was positively correlated with the number of kernels per row, number of rows per ear, ear length, and 1000-grain weight. Also, based on these traits, heatmap, and hierarchical clustering segregated the maize hybrids into distinct clusters (Figure 7B). Hybrids like IL1×IL2 and SC-131 exhibited the highest trait values (depicted in blue), whereas IL3×IL7, IL4×IL5, and IL4×IL7 showed the lowest (represented in red).

## 4. Discussion

Excessive nitrogen fertilizer use leads to economic and environmental damage, including soil acidification, water and air pollution, and various health-related issues. In this regard, developing high-yielding hybrids that adapt to low-nitrogen conditions is crucial for ensuring food security, enabling cultivation in areas with limited nitrogen availability, and minimizing environmental pollution. Studying genetic diversity and combining abilities is critical in developing maize hybrids tolerant to low nitrogen conditions. This approach enables breeders to identify and integrate the most resilient and efficient traits for enhanced performance in nutrient-poor environments. The observed significant variations among the evaluated hybrids indicate sufficient variability among the tested hybrids, which allows the possibility of selecting hybrids with outstanding performance under low N and recommended conditions. In this context, Okunlola et al. [14], Riache et al. [45], and Makore et al. [46] depicted significant genetic diversity in various agronomic traits under both optimal and low soil nitrogen conditions. Significant H×N interaction indicates that the hybrids varied in response and ranking across contrasting N supplies. This presents opportunities to identify hybrids with a broad adaptation to different N supply conditions. This finding aligns with the observations of Mebratu et al. [47], Dosho et al. [48], and Makumbi et al. [36], who detected significant H×N for yield traits. Under low N stress, there were notable declines in all traits studied compared to conditions with recommended nitrogen levels. The decrease in plant height under nitrogen-deficient conditions can be linked to inhibited cell division, cell elongation, and overall growth processes due to the reduced synthesis of critical biomolecules such as proteins and nucleic acids [49]. The significant reduction in yield-related traits under low-N conditions can be attributed to stunted plant growth, lowered photosynthetic efficiency, disrupted enzyme activities and hormonal balance, as well as diminished capacity for grain filling, which in turn reduced the 1000-kernel weight and overall grain yield [50,51,52]. Likewise, Chen et al. [16], Akhtar et al. [53], Ertiro et al. [54], Abe et al. [55] reported substantial reductions in grain yield of maize hybrids under low-N conditions.

Molecular markers successfully evaluated genetic variability and diversity within maize inbred lines [3,56,57]. These markers could help maize breeders identify and select inbred lines with the desired traits for producing high-performing hybrids. This study highlighted the level of genetic variability among the inbred lines using SSR markers. The findings indicated that the number of alleles per locus varied from 2 to 6, with an average locus. These results align with the studies conducted by Menkir et al. [58], Wegary et al. [59], and Kamara et al. [60], which reported average allele counts of 3.0, 4.2, and 4.2 alleles per locus in the same order. These figures were lower than the reported averages of 7.7 and 7.4 alleles per locus by Reif et al. [61] and Mathiang et al. [3]. The variations in the number of alleles/loci observed among the published reports could be due to various factors, including the population size and the types of SSR markers utilized. The PIC value evaluated the ability of SSR loci to detect genetic differences among inbreds based on their genetic relations. In the present study, the average PIC value was 0.54, comparable to or greater than the average PIC values reported in previous maize studies using SSR markers [62,63]. The SSR markers phi-10841, phi-024, umc-1014, 1umc-2332, and umc-1033 showed a greater ability to distinguish parental genotypes owing to their high PIC > 0.60. These identified markers might be useful for future genetic studies in maize. The high genetic diversity value of 0.73 observed through the SSR analysis in this study is indicative of a significant number of genetic variations among the assessed inbreds. The applied SSR markers categorized the inbred lines into three distinct groups. Crossing genetically diverse parents from these different groups makes it possible to create hybrids that exhibit complementary traits and enhanced heterosis [64]. Additionally, the insights gained from the cluster analysis could help reduce the number of crosses that need to be evaluated in the field.

Developing low-N tolerant and high-performing hybrids is primarily determined by selecting appropriate inbred lines. Therefore, identifying diverse inbred lines with favorable alleles is critical to ensure their transmission to the offspring [65]. The observed desirable GCA effects of the inbreds IL1 and IL3 for silking date suggest their usefulness for enhancing earliness. The inbred IL7 was predisposed to be a valuable combiner for decreasing plant height and ear height, which is crucial for boosting tolerance to lodging. Shorter hybrids might be more efficient in partitioning photosynthates to develop ears than increasing biomass. The results identified the parental line IL8 as a good combiner for lower ear placement and early maturity under both nitrogen treatments. This suggests its utility in developing early genotypes with desirable ear placement under recommended and low N conditions. Additionally, IL1, IL2, and IL3 inbred lines exhibited highly favorable GCA for yield traits under low N conditions. These lines could serve as valuable genetic resources for enhancing maize yield under nitrogen stress by transferring favorable alleles to their progeny. These inbreds are also well-suited for creating superior offspring when crossed with other inbreds under low-N stress conditions [46,53]. Desirable hybrids were identified by assessing specific combining ability (SCA) effects. Several hybrids demonstrated positive SCA effects for at least one trait under both low N and recommended conditions. Notably, the cross combinations IL1×IL4, IL2×IL5, IL2×IL6, and IL5×IL7 under low N conditions, along with IL1×IL3, IL1×IL6, IL3×IL5, and IL4×IL7 under both conditions, were identified as superior specific combiners for breeding high-yielding maize hybrids. These promising hybrids can be strategically integrated into maize breeding programs to improve yield potential and overall productivity under varying nitrogen levels. Interestingly, these hybrids often result from crosses between parents showing good and poor GCA, likely due to one parent providing strong additive effects and the other contributing beneficial epistatic interactions [66]. Both additive and non-additive gene actions significantly influenced the inheritance of the agronomic traits studied, as evidenced by the notable GCA and SCA effects. The GCA/SCA ratio exceeded one for all traits measured, indicating that additive gene action plays a crucial role in governing the inheritance of these traits. This finding aligns with the research by Owusu et al. [67], Ribeiro et al. [38], Badu-Apraku et al. [68], and Okunlola et al. [14], who also observed additive genetic variance to be predominant in the expression of grain yield across different nitrogen levels. However, this stands in contrast to the studies of Betran et al. [69], Worku et al. [70], and Makinde et al. [71], which highlighted the dominance of non-additive gene action in the genetic control of grain yield under similar conditions.

## 5. Conclusions

The applied markers revealed substantial genetic diversity among the evaluated parental inbred lines, offering valuable insights for maize breeding programs by identifying distinct inbreds suitable for crossbreeding. Significant differences were observed across nitrogen levels, hybrids, and their interactions for all assessed traits. The inbred line IL3 was recognized as an effective combiner for developing early maturing genotypes with lower ear placement. Additionally, inbreds IL1, IL2, and IL3 were identified as superior combiners for enhancing grain yield and its associated traits under both low-N and recommended conditions. The hybrids IL1×IL4, IL2×IL5, IL2×IL6, and IL5×IL7 were pinpointed as specific good combiners for boosting grain yield and related traits under low-N stress. Strong positive correlations existed between grain yield and agronomic traits such as ear length, plant height, number of rows per ear, and number of kernels per row, underscoring their potential for indirect selection in early breeding generations, especially under low-N stress conditions due to their ease of measurement.

Further directions could deepen the understanding of maize genetics and breeding and align research efforts with practical agricultural needs and environmental sustainability, expanding genetic diversity analysis by incorporating more inbred lines from different geographic regions. Moreover, the performance of developed hybrids across various environmental conditions could be assessed over multiple growing seasons to explore their resilience to nutrient deficiencies and stability.

## Figures and Tables

**Table 1:** Genetic distance among the assessed inbred lines using SSR markers.

Parent	IL1	IL2	IL3	IL4	IL5	IL6	IL7
IL1	0.00						
IL2	0.43	0.00					
IL3	0.71	0.78	0.00				
IL4	0.53	0.63	0.71	0.00			
IL5	0.88	0.88	0.83	0.88	0.00		
IL6	0.71	0.84	0.78	0.84	0.83	0.00	
IL7	0.65	0.72	0.72	0.79	0.74	0.65	0.00

**Table 2:** Mean squares from ordinary analysis and combining ability for all the studied traits.

**Source of Variation**	**df**	**Days to** **Silking**	**Plant Height** **(cm)**	**Ear Height** **(cm)**	**Ear Length** **(cm)**
Nitrogen (N)	1	1334 **	174,141 **	66,590 **	383.5 *
Error a	4	12.42	3854	60.08	44.65
Hybrids (H)	20	50.37 **	1705 **	978.7 **	18.50 **
GCA	6	58.91 **	1755 **	1374 **	34.64 **
SCA	14	46.71 **	1684 **	809.3 **	11.58 **
H×N	20	6.31 *	299.9 *	511.9 **	10.52 **
GCA×N	6	4.15	388.8 *	454.3 **	8.71 **
SCA×N	14	7.24 *	261.8	536.6 **	11.29 **
Error b	80	3.63	156.4	90.85	0.75
GCA/SCA		1.26	1.04	1.70	2.99
GCA×N/SCA×N		0.57	1.48	0.85	0.77
**SOV**	**df**	**Number of Rows per Ear**	**Number of Kernels per Row**	**Thousand Kernel Weight (g)**	**Grain Yield** **(t/ha)**
Nitrogen (N)	1	118.1 **	1551 **	211,396 **	141.6 **
Error a	4	1.21	1.27	200.3	1.06
Hybrids (H)	20	6.89 **	114.0 **	5305 **	13.03 **
GCA	6	11.30 **	230.4 **	9144 **	33.48 **
SCA	14	5.00 **	64.09 **	3660 **	4.27 **
H×N	20	1.28 *	35.12 **	769.2 **	1.72 **
GCA×N	6	1.42	42.69 *	976.5 **	3.21 **
SCA×N	14	1.22 *	31.88 *	680.4 **	1.08 **
Error b	80	0.66	14.13	149.1	0.15
GCA/SCA		2.26	3.60	2.50	7.84
GCA×N/SCA×N		1.16	1.34	1.44	2.97

* and ** signify *p*-values less than 0.05 and 0.01, in the same order; df stands for degrees of freedom.

**Table 3:** General combining ability (${\hat{g}}_{i}$) effects of the seven inbreds for evaluated agronomic traits under low and recommended nitrogen levels.

**Line**	**Days to Silking**		**Plant Height (cm)**		**Ear Height (cm)**		**Ear Length (cm)**	
	**Low N**	**Recommended** **N**	**Low** **N**	**Recommended** **N**	**Low** **N**	**Recommended** **N**	**Low N**	**Recommended** **N**
IL1	−1.22 *	−2.10 **	6.66	8.86 **	7.58 **	6.74 **	1.22 **	1.78 **
IL2	−0.89	0.44	11.53 **	9.60 **	5.45 *	16.87 **	0.80 **	0.84 **
IL3	−2.23 **	−1.70 **	−1.76	7.10 *	−7.75 **	−5.89 *	1.09 **	0.22
IL4	0.18	−0.16	−4.00	−7.49 **	2.65	−3.01	−1.10 **	0.51 *
IL5	1.58 **	0.97 *	−1.74	2.94	−6.69 **	−1.67	−1.02 **	−2.08 **
IL6	0.85	0.97 *	1.33	−12.09 **	5.11 *	−7.93 **	0.48 *	−0.69 **
IL7	1.71 **	1.57 **	−12.03 **	−8.92 **	−6.35 **	−5.13 *	−1.48 **	−0.58 *
LSD (0.05) gi	0.95	0.89	6.67	5.34	4.60	4.61	0.39	0.45
LSD (0.01) gi	1.27	1.19	8.93	7.14	6.15	6.17	0.52	0.60
**Inbred Line**	**No. of Rows per Ear**		**No. of Kernels per Row**		**1000-Kernel Weight (g)**		**Grain Yield (t/ha)**	
	**Low** **N**	**Recommended** **N**	**Low** **N**	**Recommended** **N**	**Low** **N**	**Recommended N**	**Low N**	**Recommended** **N**
IL1	0.54 *	0.89 **	2.75 **	4.80 **	10.63 **	17.52 **	0.95 **	1.26 **
IL2	0.68 **	0.22	3.82 **	1.67 *	32.63 **	15.52 **	1.61 **	1.07 **
IL3	0.34	0.35	2.22 *	0.00	9.16 **	14.86 **	0.73 **	0.87 **
IL4	−1.39 **	−0.85 **	−0.91	0.07	−17.77 **	−13.14 **	−1.36 **	−0.68 **
IL5	−0.06	−0.71 **	−1.58	−1.00	−0.50	−14.81 **	−0.52 **	−1.23 **
IL6	−0.26	0.02	−0.25	−2.93 **	−2.84	−2.14	0.07	−0.63 **
IL7	0.14	0.09	−6.05 **	−2.60 **	−31.30 **	−17.81 **	−1.48 **	−0.65 **
LSD (0.05) gi	0.41	0.37	2.03	1.57	6.37	5.39	0.18	0.19
LSD (0.01) gi	0.55	0.50	2.72	2.10	8.52	7.21	0.24	0.26

* and ** signify *p*-values less than 0.05 and 0.01 in the same order.

**Table 4:** Specific combining ability (SCA) effects for twenty-one F1 maize hybrids on agronomic traits under different nitrogen levels.

**Cross**	**Days to Silking**		**Plant Height (cm)**		**Ear Height (cm)**		**Ear Length (cm)**	
	**Low** **N**	**Recommended N**	**Low** **N**	**Recommended N**	**Low** **N**	**Recommended N**	**Low** **N**	**Recommended N**
IL1×IL2	2.98 **	2.02 *	−3.14	13.68 *	21.73 **	−7.50	1.54 **	−0.44
IL1×IL3	0.31	3.82 **	12.82	3.40	19.93 **	21.26 **	−1.04 **	1.59 **
IL1×IL4	−2.42 *	−1.38	15.06 *	1.00	−1.13	6.72	2.51 **	−1.74 **
IL1×IL5	1.84	−0.18	27.12 **	20.56 **	−18.47 **	11.72 *	−1.50 **	−0.55
IL1×IL6	−0.09	2.16 *	−22.28 **	−4.40	−5.60	−15.36 **	−1.40 **	1.79 **
IL1×IL7	−2.62 **	−6.44 **	−29.58 **	−34.24 **	−16.47 **	−16.83 **	−0.10	−0.65
IL2×IL3	−0.02	−3.04 **	−1.05	−12.33 *	−17.93 **	−16.20 **	−0.46	−0.98 *
IL2×IL4	0.91	0.09	5.19	−5.74	−7.00	4.25	−1.84 **	−4.08 **
IL2×IL5	0.51	1.62	1.92	11.33 *	−8.33	4.92	0.19	1.79 **
IL2×IL6	−6.09 **	−4.04 **	−12.14	−5.14	14.20 **	16.50 **	0.82 *	2.02 **
IL2×IL7	1.71	3.36 **	9.22	−1.81	−2.67	−1.96	−0.25	1.69 **
IL3×IL4	−2.09 *	−2.11 *	−13.52 *	−9.24	−1.80	15.61 **	0.11	2.15 **
IL3×IL5	−2.82 **	−1.91 *	4.88	12.32 *	10.20 *	−12.39 **	1.70 **	−2.12 **
IL3×IL6	4.24 **	2.42 **	12.15	5.16	−12.27 **	−6.07	2.00 **	0.08
IL3×IL7	0.38	0.82	−15.29 *	0.68	1.87	−2.20	−2.30 **	−0.72
IL4×IL5	1.11	0.22	−26.88 **	−13.08 *	−5.53	−6.54	−1.51 **	1.42 **
IL4×IL6	3.18 **	2.56 **	10.39	6.95	6.67	−5.28	0.38	−0.21
IL4×IL7	−0.69	0.62	9.75	20.11 **	8.80	−14.75 **	0.35	2.47 **
IL5×IL6	−1.56	−2.24 *	−10.54	−24.48 **	5.33	−11.62 *	−1.49 **	−0.72
IL5×IL7	0.91	2.49 **	3.48	−6.66	16.80 **	13.92 **	2.61 **	0.18
IL6×IL7	0.31	−0.84	22.42 **	21.91 **	−8.33	21.84 **	−0.30	−2.97 **
LSD 5% (sij)	1.87	1.76	13.16	10.52	9.07	9.09	0.76	0.88
LSD 1% (sij)	2.50	2.35	17.60	14.08	12.13	12.17	1.02	1.18
**Cross**	**No. of Rows per Ear**		**No. of Kernels per Row**		**1000-Kernel Weight (g)**		**Grain Yield (t/ha)**	
	**Low** **N**	**Recommended** **N**	**Low-N**	**Recommended** **N**	**Low** **N**	**Recommended** **N**	**Low-N**	**Recommended** **N**
IL1×IL2	1.18 **	1.36 **	0.44	0.87	13.82 *	13.78 *	0.08	0.48 *
IL1×IL3	1.18 **	0.89 *	−0.29	1.87	−26.04 **	−15.56 **	0.56 **	0.67 **
IL1×IL4	0.58	0.09	−1.16	0.13	−20.44 **	12.44 *	0.64 **	0.00
IL1×IL5	−1.42 **	−0.38	2.51	−0.80	20.29 **	24.11 **	−1.55 **	−0.75 **
IL1×IL6	−0.56	−0.44	2.18	2.13	19.29 **	11.44 *	0.77 **	0.56 **
IL1×IL7	−0.96 *	−1.51 **	−3.69	−4.20 **	−6.91	−46.22 **	−0.49 **	−0.96 **
IL2×IL3	−0.62	−1.11 **	−3.02	0.00	−1.38	−3.56	0.13	−1.10 **
IL2×IL4	−0.22	1.42 **	−7.22 **	−4.40 **	18.89 **	14.44 **	−1.13 **	−0.84 **
IL2×IL5	0.44	−1.38 **	2.11	0.00	−38.38 **	−23.89 **	0.66 **	0.16
IL2×IL6	−1.36 **	−0.78 *	2.44	4.60 **	0.62	0.11	0.79 **	0.22
IL2×IL7	0.58	0.49	5.24 *	−1.07	6.42	−0.89	−0.52 **	1.08 **
IL3×IL4	0.11	−0.71	−0.62	2.60	39.02 **	35.11 **	−0.26	1.04 **
IL3×IL5	0.44	0.82 *	4.04 *	4.00 *	15.09 *	6.78	1.16 **	0.85 **
IL3×IL6	0.31	0.42	−1.29	−6.07 **	7.42	−2.56	−0.37 *	−0.09
IL3×IL7	−1.42 **	−0.31	1.18	−2.40	−34.11 **	−20.22 **	−1.21 **	−1.37 **
IL4×IL5	−0.16	−0.31	1.18	0.60	−27.98 **	−36.89 **	−0.39 *	−0.65 **
IL4×IL6	0.04	−0.04	5.18 *	−4.13 *	−12.31	−42.89 **	−0.18	−0.69 **
IL4×IL7	−0.36	−0.44	2.64	5.20 **	2.82	17.78 **	1.33 **	1.13 **
IL5×IL6	0.04	0.16	−6.49 **	−1.40	−7.91	7.11	−0.88 **	0.13
IL5×IL7	0.64	1.09 **	−3.36	−2.40	38.89 **	22.78 **	1.01 **	0.26
IL6×IL7	1.51 **	0.69	−2.02	4.87 **	−7.11	26.78 **	−0.12	−0.14
LSD 5% (sij)	0.80	0.74	4.01	3.09	12.56	10.63	0.35	0.38
LSD 1% (sij)	1.08	0.99	5.37	4.14	16.80	14.22	0.47	0.51

* and ** signify *p*-values less than 0.05 and 0.01 in the same order

## Data Availability

The data presented in this study are available upon request from the corresponding author.

## Supplementary Materials

The following supporting information can be downloaded at: https://www.mdpi.com/article/10.3390/life14050641/s1, Table S1: Code, name, pedigree and source of the seven maize inbred lines; Table S2: Some physical and chemical soil characteristics of the experimental sites during 2022 and 2023 growing seasons; Table S3: List of SSR primers and their sequences used in the present study; Table S4: Mean squares from ordinary analysis and combining ability for the studied traits under low and recommended nitrogen levels.
