# Supplementary material for: Molecular Diversity and Combining Ability in Newly Developed Maize Inbred Lines under Low-Nitrogen Conditions
Source: Life (Basel). 2024 May 17;14(5):641. doi: 10.3390/life14050641 (PMC11122723; doi:10.3390/life14050641)
Supplement: Supplementary file 1 [file life-14-00641-s001.zip › life-3005237-supplementary.pdf]

Table S1. Code, name, pedigree and source of the seven maize inbred lines

Parent code	Pedigree	Source
I.L. 1	Locally developed	Egypt
I.L. 2	Locally developed	Egypt
I.L. 3	Locally developed	Egypt
I.L. 4	Locally developed	Egypt
I.L. 5	[EMSR]#B#bF101sr-2-1-sr-3-2-4-b-b	CIMMYT-Mexico
I.L. 6	[EV7992#/EVPO44-SRBC3]#bF37sr-2-3-sr-3-5-2-b-b	CIMMYT-Mexico
I.L. 7	[EV7992#/EVPO44-SRBC3]#bF37sr-2-3-sr-2-4-3-b-b	CIMMYT-Mexico

Table S2. Some physical and chemical soil characteristics of the experimental sites during 2022 and 2023 growing seasons.

Properties	2022	2023
Soil texture	Clay	Clay
Sand (%)	14.90	13.20
Silt (%)	32.50	33.20
Clay (%)	52.60	53.60
pH (1: 2.5 water suspension)	8.31	8.11
EC (dSm^{-1})	3.55	3.65
Organic matter (%)	1.85	1.76
Available Nitrogen (N) (mg kg^{-1})	38.56	36.75
Available phosphorus (P) (mg kg^{-1})	9.42	10.66
Available potassium (K) (mg kg^{-1})	260.63	245.80

Table S3. List of SSR primers and their sequences used in the present study.

Marker	Forward primer	Reverse primer
phi308707	GCAACAAGATCCAGCCGAT	GTCGCCCTCATATGACCTTC
phi96100	AGGAGGACCCCAACTCCTG	TTGCACGAGCCATCGTAT
phi453121	ACCTTGCCTGTCCTTCTTTCT	CAAGCAAGACTTTTGATCAGCC
umc2038	ACAGAAACCAATGCATGTGATGAG	ACAGAAACCAATGCATGTGATGAG
phi024	ACTGTTCCACCAAACCAAGCCGAGA	AGTAGGGTTGGGGATCTCCTCC
Umc1014	GAAAGTCGATCGAGAGACCCTG	CCCTCTCTTCACCCCTTCCTT
umc2332	GTCGGAGAAGGAGCTACTGAGCTA	CACAGGTACGTCTGGATGCTGT
phi015	GCAACGTACCGTACCTTTCCGA	ACGCTGCATTCAATTACCGGGAAG
umc1033	CTTCTTCGTAAAGGCATTTTGTGC	GTGCGGGATTCCTTAGTTTGC
phi108411	CGTCCCTTGGATTTCGAC	CGTACGGGACCTGTCAACAA
phi301654	GAATGCATGCTTTTCAAGGAC	CGCACAGAGAGCAGAACG

Table S4: Mean squares from ordinary analysis and combining ability for the studied traits under low and recommended nitrogen levels.

SOV	df	Days to silking		Plant height (cm)		Ear height (cm)		Ear length (cm)	
		Low N	Recommended N	Low N	Recommended N	Low N	Recommended N	Low N	Recommended N
Hybrids (H)	20	12.34**	16.68**	996.17**	1009.09**	642.67**	847.84**	12.34**	16.68**
GCA	6	6.68**	7.78**	288.32**	426.29**	221.22**	388.12**	6.68**	7.78**
SCA	14	3.02**	4.61**	350.80**	297.82**	211.23**	237.40**	3.02**	4.61**
Error	40	0.64	0.86	190.74	122.04	90.57	91.13	0.64	0.86
GCA/SCA		2.21	1.69	0.82	1.43	1.05	1.63	2.21	1.69
SOV	df	No. of rows per ear		No. of kernels per row		1000-kernel weight (g)		Grain yield (t/ha)	
		Low N	Recommended N	Low N	Recommended N	Low N	Recommended N	Low N	Recommended N
Hybrids (H)	20	4.32**	3.85**	85.98**	63.13**	3353.56**	2720.75**	8.28**	6.47**
GCA	6	2.41**	1.83**	55.88**	35.16**	2138.04**	1235.53**	6.97**	5.26**
SCA	14	1.02**	1.05**	17.00**	15.00**	680.63**	766.08**	0.96**	0.83**
Error	40	0.71	0.60	17.72	10.54	173.79	124.44	0.14	0.16
GCA/SCA		2.35	1.74	3.29	2.34	3.14	1.61	7.28	6.36

** signify p-values less 0.01
